# Asparagus Fructans as Emerging Prebiotics

**DOI:** 10.3390/foods12010081

**Published:** 2022-12-23

**Authors:** Amel Hamdi, Isabel Viera-Alcaide, Rafael Guillén-Bejarano, Rocío Rodríguez-Arcos, Manuel Jesús Muñoz, Jose Manuel Monje Moreno, Ana Jiménez-Araujo

**Affiliations:** 1 Instituto de la Grasa, Consejo Superior de Investigaciones Científicas (CSIC), Pablo de Olavide Universitary Campus, Building 46, Carretera de Utrera Km 1, 41013 Seville, Spain; 2 Molecular Biology and Biochemical Engineering Department, Centro Andaluz de Biología del Desarrollo (CABD), University Pablo de Olavide (UPO), CSIC/UPO/JA, Carretera de Utrera Km 1, 41013 Seville, Spain

**Keywords:** asparagus by-product, fructan extract, commercial fructans, degree of polymerization, physicochemical characteristics, antioxidant activity, FTIR analysis, prebiotic properties, *C. elegans* models, circular economy

## Abstract

Commercial fructans (inulin and oligofructose) are generally obtained from crops such as chicory, Jerusalem artichoke or agave. However, there are agricultural by-products, namely asparagus roots, which could be considered potential sources of fructans. In this work, the fructans extracted from asparagus roots and three commercial ones from chicory and agave were studied in order to compare their composition, physicochemical characteristics, and potential health effects. Asparagus fructans had similar chemical composition to the others, especially in moisture, simple sugars and total fructan contents. However, its contents of ash, protein and phenolic compounds were higher. FTIR analysis confirmed these differences in composition. Orafti^®^GR showed the highest degree of polymerization (DP) of up to 40, with asparagus fructans (up to 25) falling between Orafti^®^GR and the others (DP 10–11). Although asparagus fructan powder had a lower fructan content and lower DP than Orafti^®^GR, its viscosity was higher, probably due to the presence of proteins. The existence of phenolic compounds lent antioxidant activity to asparagus fructans. The prebiotic activity in vitro of the four samples was similar and, in preliminary assays, asparagus fructan extract presented health effects related to infertility and diabetes diseases. All these characteristics confer a great potential for asparagus fructans to be included in the prebiotics market.

## 1. Introduction

Fructans, present in nature as storage polysaccharides in plants, are widely used in the food, nutraceutical and pharmaceutical industries. They are basically made up of fructose units linked to the glucose moiety of a sucrose molecule. Due to structural differences, these polymers can be classified into five groups: (i) inulin-type fructans, based on a 1-kestose structure; (ii) levan-type fructans, from a 6-kestose molecule; (iii) graminans, with branched inulin or levan structure; (iv) inulin neoseries, synthesized from a neo-kestose molecule, and (v) mixed-type levans, from a neo-levan structure. Fructans have applications as functional food and as prebiotic nutrients [1,2]. They are resistant to digestion in the upper portion of the human intestinal tract due to the β configuration of anomeric C-2 but they can be fermented by some beneficial colonic bacteria, popularly known as probiotics [3]. This fermentation process brings beneficial health effects, such as enhancement in the intestinal absorption of calcium, magnesium and iron [4], improvement in obesity prevention or control, blood glucose level, and lipid metabolism by the production of short-chain fatty acids [5], stimulation of the immune system [6] and reduction in the chances of cancer and allergic reactions [7].

In addition to their interesting nutritional and health benefits, fructans are also used in food formulations for their techno-functional properties such as a replacement for sugar in low-calorie food [8]. Due to their gelling capacity, they can easily be used as a fat substitute in food preparations. The product formulated with fructans instead of fat has a similar mouthfeel, with an appropriate creamy and consistent texture. Considered dietary fiber, they also have a low-calorie content, about 1–1.5 kcal per gram [9]. Fructans have synergistic effects with most gelling agents, e.g., gelatin, alginate, k- and i-carrageenans, gellan gum and maltodextrins. In addition, they also work as foam and emulsion stabilizers. Therefore, they can be included in the formulation of aerated desserts, ice creams, table spreads and sauces, replacing synthetic additives. Related to their gelling characteristics, fructans have humectant properties, affecting boiling and freezing points and reducing water activity. In this way, they can also act as an antimicrobial agent [10].

Due to their wide distribution in the plant kingdom and their varied applications in food technology, the extraction, isolation and characterization of fructans are gaining interest. The chemical characteristics of fructans depend on the species and also vary with the environmental conditions and developmental stage of plants [11]. Currently, chicory root (*Cichorium intybus* L.) is the main feedstock for the industrial production of inulin in Europe, with 15–20% of inulin on a fresh weight basis. Jerusalem artichoke (*Helianthus tuberosus* L.) and agave (*Agave tequilana* Weber) are also considered natural sources of inulin in other world zones, especially in North America. Jerusalem artichoke and its by-products contain a large amount of fructans (11–14% fresh weight basis) [12] and the remaining feedstock is utilized for the production of ethanol [13]. Fructans are also extracted from agave (higher than 37% on a fresh weight basis), which is often utilized as a bulking agent and can serve as a sugar replacement [14].

Recent studies conducted by Viera-Alcaide et al. [15], Weerasingha et al. [16], Sun et al. [17] and Mudannayake et al. [8] found that the roots and the rhizome of some *Asparagus* species such as, *A. officinalis*, *A. falcatus*, *A. racemosus* and *A. cochinchinensis* have the potential to be used as alternative inulin sources. The global cultivation area of different *Asparagus* species is higher than that of chicory and Jerusalem artichoke [8,16,18]. Compared to the commercial fructan sources, *A. falcatus*, *A. racemosus* and *A. officinalis* have shown 17.74, 11.83 and 15.3% of inulin, respectively, in the roots on a fresh weight basis [8,16,18].

The addition of fructans to different food systems is a worldwide practice. However, asparagus fructans and their prebiotic properties remain almost unknown. Weerasingha et al. [16] added *A. officinalis* fructan as an ingredient in the production of yogurt and concluded that its addition did not change the nutritional quality of this probiotic food. After a sensory analysis, asparagus fructan-added yogurts scored higher than plain yogurts in all the evaluated attributes and also improved their physicochemical characteristics. Moreover, the prebiotic properties of asparagus fructans were stated in this same study, since the viability of yogurt probiotic bacteria (*Bifidobacterium bifidum, Lactobacillus bulgaricus* ssp. bulgaricus and *Streptococcus thermophilus*) increased proportionally to the fructan concentration during the storage period. In another study [17], a purified fructan from the roots of *A. cochinchinensis* was subjected to in vitro fermentation by human fecal microbiota. As the main physicochemical effects, a decrease in the pH of the culture medium was detected, together with an increase in the short-chain fatty acid concentration, especially that of acetic, propionic, i-valeric and *n*-valeric acids. Some changes were also observed in the microbiota composition after 24 h fermentation: the beneficial genus *Prevotella, Megamonas*, and *Bifidobacterium* increased proportionally, while the genus *Haemophilus*, related to respiratory and nervous diseases, decreased. These results pointed to a health-promoting effect linked to asparagus fructan consumption.

As recent research has demonstrated good perspectives for the use of asparagus fructans in food and nutraceuticals applications, and considering that an agro-food by-product such as roots and rhizomes from asparagus cultivation are the feedstock for their isolation, there is increasing interest in developing this alternative fructan source. In our previous work, we established the concept of roots and rhizomes of asparagus as a valuable source of fructans [15]. As a continuation, this paper aimed to study the chemical composition, physicochemical characteristics, and in vitro prebiotic activity of the asparagus root fructans and three commercial ones in order to verify the capability of asparagus fructans in contrast to others currently on the market, and to determine their potential health effects using *Caenorhabditis elegans* as a model organism.

## 2. Materials and Methods

### 2.1. Plant Material

Underground asparagus organs were harvested during autumn, 2019, at the end of the harvest season, at different asparagus exploitation fields in Huétor-Tájar, Granada (Spain). The rhizomes and roots were sent to the Instituto de la Grasa labs, washed to remove soil and other foreign material, and left to dry at room temperature. The samples were chopped with an IKRA Mogatec shredder model EG 2500 in order to obtain a homogeneous raw material for fructan extraction and frozen at −20 °C.

### 2.2. Fructan Extraction from Asparagus Roots

Fructans were extracted as previously described [15]: 500 g of frozen sample were homogenized with 4 L of boiling water and placed in a shaking water bath set at 80 °C and 60 rpm for 30 min. Afterward, the sample was filtered through filter paper and the slurry was extracted again under the same conditions with 2 L of hot water. Both filtrates were mixed and stored at −20 °C.

### 2.3. Purification of Asparagus Fructans by Adsorption Chromatography

A glass column was filled with 100 mL (3 × 15 cm) of Diaion^®^ HP20, a synthetic adsorbent resin of the highly porous type obtained from Vivaqua International S.L. (Barberà del Vallès, Spain). The resin was activated with 96% ethanol and then equilibrated with distilled water. In total, 1 L of extract was loaded into the column at a flow rate of 2-bed volumes/h and the column was washed with 200 mL water, 400 mL of 20% ethanol, and 400 mL of 80% ethanol. The non-retained fraction, water and 20% ethanol washes were put together, concentrated under vacuum and freeze-dried. The resultant pale-brown solid was considered to be purified asparagus fructans (PAF).

### 2.4. Commercial Fructan Sources

Orafti^®^GR from Beneo GmbH (Mannheim, Germany) was kindly supplied by Ferrer Alimentación S.A. (Barcelona, Spain). Chicory inulin from SaludViva (Elche, Spain) and agave inulin (OligofructineTM) from Tentorium Energy S.L. (Ulldecona, Spain) were purchased from local health food stores.

### 2.5. Determination of Chemical Composition

#### 2.5.1. Moisture

Aliquots of PAF and commercial inulins (1 g × 3) were dried in an infrared moisture analyzer (Ohaus, MB45) until constant weight. The result is expressed as a g/100 g fresh weight.

#### 2.5.2. Simple Sugar Composition

Solutions of 1 mg/mL of the different samples were analyzed in a Dionex (Sunnyvale, CA, USA) high-performance anion-exchange chromatograph (HPAEC) using a Carbopac PA-10 column (4 × 250 mm, 10 μm) in combination with a Carbopac PA guard column (4 × 50 mm, 10 μm) as described by Jaramillo-Carmona et al. [19]. The mobile phase consisted of 100 mM NaOH (eluent A) and 100 mM NaOH and 700 mM sodium acetate (eluent B). The elution conditions were as follows: 0–15 min, 100% A (re-equilibration); injection at 15 min and start acquisition at 16 min; linear gradient over 45 min to 25% A, 75% B. The flow rate was maintained at 0.9 mL/min. A Dionex pulsed electrochemical detector in the pulsed amperometric detection (PAD) mode was used. Calibration curves of standard glucose, fructose and sucrose were used for the quantification of mono- and disaccharides.

#### 2.5.3. Ash

Ashes were determined by incinerating samples in a muffle furnace Nabertherm model B180 (Bremen, Germany) at 550 °C until white ash was obtained.

#### 2.5.4. Proteins

The concentration of protein was determined as % nitrogen content × 6.25 by elemental microanalysis using a Leco CHNS932 analyzer (St. Joseph, MI, USA).

#### 2.5.5. Fructans

The fructan content in each product was analyzed by the K-FRUC assay Kit from Megazyme (Bray, Ireland). The complete method is described in detail on the Megazyme website [20]. The results were expressed as g/100 g fresh weight product.

#### 2.5.6. Phenols

Solutions of 10 mg/mL of each sample were analyzed by HPLC [21]. Briefly, the analyses were carried out using a Jasco-LC-Net II ADC liquid chromatograph system equipped with a diode array detector (DAD). Phenols were separated using a Mediterranea Sea C18 reverse-phase analytical column (25 cm length × 4.6 mm i.d., 5 μm particle size; Teknokroma, Barcelona, Spain). The gradient profile was formed using solvent A (water with 1% formic acid) and solvent B (acetonitrile with 1% formic acid): from 0% B to 20% B for the first 20 min, to 21% B over the next 8 min, maintained at 21% B for 2 min, and then to 30% B over the next 10 min, and to 100% over the next 5 min, and finally maintained at 100% B for 5 min. The flow rate was 1 mL/min and the column temperature was 30 °C. Spectra from all peaks were recorded in the 200–600 nm range and the chromatograms were acquired at 360 nm. The quantification of individual phenols was performed using an eight-point regression curve in the range of 0–250 μg on the basis of standards.

### 2.6. pH

Solutions of 100 mg/mL were made from PAF and other commercial samples to determine pH values. A pHmeter Mettler-Toledo (Barcelona, Spain) model FiveEasy was used for the measurements.

### 2.7. Antioxidant Activity

The antioxidant activity was measured against DPPH· free radical [22]. The efficient concentration (EC_50_), which represents the amount of antioxidants necessary to decrease the DPPH· initial absorbance by 50%, was calculated from a calibration curve by linear regression for each sample. The activity was expressed as millimoles of Trolox equivalent (TE) per kilogram of sample by means of a dose–response curve for Trolox.

### 2.8. Degree of Polymerization of Fructans

The samples were analyzed in a Dionex (Sunnyvale, CA, USA) high-performance anion-exchange chromatograph (HPAEC) under similar conditions to those described for simple sugar determination. The elution conditions were as described in Viera-Alcaide et al. [15]: 0–15 min, 100% A (re-equilibration); injection at 15 min and start acquisition at 16 min; linear gradient over 55 min to 20% A, 80% B. The flow rate was maintained at 0.9 mL/min. The identification of the different DP was made using Orafti^®^GR. 

### 2.9. Viscosity

Viscosity measurements were taken with a Cannon-Fenske viscometer, series 150. A set of solutions of the four inulin samples (5–30% *w*/*v*) were prepared at 80 °C with continuous stirring. The viscosity assays were developed at three temperatures 80, 20 and 4 °C in a water bath with a thermostat. The time the sample took to pass between the two marks in the viscometer (×3), in seconds, is used to calculate the dynamic viscosity using the following formula:

η = C × t × ρ

where η is the dynamic viscosity, expressed in cP; C is the viscometer constant at the assay temperature; t is the time to pass in seconds; and ρ is the density of the sample at the assay temperature in g/mL.

### 2.10. Oil Holding Capacity (OHC)

OHC was measured as previously described [23], with some modifications: 500 mg of each sample, in triplicate, were weighed into 15 mL centrifuge tubes and 5 g of sunflower oil was added to each tube. The tubes were stirred and left to settle for 60 min. Afterward, the samples were centrifuged in a Digtor 22-R centrifuge from Orto-Alresa (Daganzo de Arriba, Spain) set at 3122× *g*, 20 °C, for 20 min. After centrifugation, the tubes were placed upside down for 30 min to rinse the non-retained oil. The tubes were then weighed and the retained oil was calculated by weight difference. OHC was expressed as g oil/100 g inulin.

### 2.11. Color

The color determinations of the four inulin samples were measured using a color measurement spectrophotometer BYK-Gardner, Color-view model (Columbia, MD, USA) set for Hunter L* (lightness), a* (redness), b* (yellowness).

### 2.12. Fourier-Transform Infrared (FTIR) Spectroscopy

Around 2 mg of each sample was milled carefully with 200 mg of KBr using a clean and dry mortar and pestle. The dry and milled powders were pressed into discs in a Graseby hydraulic press (Specac, Orpington, UK) fitted with a 13 mm evacuable pellet die [24]. The clear KBr-fructan discs, once loaded into the holder, were exposed horizontally to the IR beam in a Bruker vertex 70 FT-IR spectrometer (Bruker Optics, Ettlingen, Germany) equipped with a deuterated triglycine sulfate (DTGS) detector. The experiments were run in duplicate to ensure consistency of the results. All the spectra were collected in the range of 5000–400 cm^−1^ by co-addition of 50 scans and a resolution of 4 cm^−1^ using weak Norton–Beer apodization [25]. The spectra were collected and manipulated with OPUS version 7.2 (Bruker Optics, Ettlingen, Germany). The differences between the FTIR spectra obtained were studied with Omnic 7.3 (Thermo Electon Inc., Madison, WI, USA).

### 2.13. Prebiotic Effect In Vitro

The prebiotic activity of the different fructan sample was evaluated against five probiotic *Lactobacillus* strains: *L. plantarum* ATCC 8014 (American Type culture collection, Manassas, VA, USA), *L. plantarum* CECT 9567 (Colección Española de Cultivos Tipo, Valencia University, Valencia, Spain), *L. reuteri* DSM 17,938 (Casen Recordati S.L., Madrid, Spain), *L. rhamnosus*, and *L. casei*. The culture media were MRS, which replaced the carbohydrate source for the different fructans at 2% (MRS+Asparagus, MRS+Orafti^®^GR, MRS+Chicory, MRS+Agave). As a control for each sample, the different strains were also incubated in MRS but only with the amount of simple sugar that comes with each fructan sample. The presented results are the difference between both assays, to discuss only the growth due to fructan fermentation. Two more control were included, MRS + 2% glucose and MRS without any carbon source [26]. In each microplate well, 10 μL of inoculum and 340 μL of medium were dosified to reach a final concentration of 10^6^ cfu/mL. The optical density (OD) at 600 nm was measured every half an hour in a SPECTROstar spectrophotometer (BMG Labtech, Ortenberg, Germany). The software includes the Reader Control and MARS data analysis interfaces, which served to measure the area under the growth curve for 36 h. All the assays were performed in triplicate.

### 2.14. C. elegans Assays

#### 2.14.1. *C. elegans* Strains and Maintenance Conditions

The *C. elegans* strains used in the study were obtained from CGC (Caenorhabditis Genetics Center, University of Minnesota, Minneapolis, MN, USA): CB1370: *daf-2(e1370ts)III* and GMC101: *dvIs100[Punc-54::amyloid-ß1-42:3′UTR unc-54 + Pmtl-2::GFP]II*. Nematode growth medium (NGM) agar plates containing Agar N◦1 (2.5% *w*/*v*, Oxoid Limited, Basingstoke, UK), Pepton N-Z-Soy(R) BL4 (0.25% *w*/*v*, Sigma Aldrich, St.-Louis, MO, USA), NaCl (0.3% *w*/*v*), cholesterol (0.0005% *v*/*v*), CaCl_2_ (1 mM), MgSO_4_ (1 mM), K_2_HPO_4_/KH_2_PO_4_ (25 mM, pH 6.0) were seeded with OP50 as a food source for all the strains, and the living temperature was set at 16 °C. The asparagus fructans extract stock solutions (6, 7 mg/mL in ultrapure water) were prepared, filtered with 0.22 µm (Syringe-driven Filters membrane CA) and stored at −20 °C. The utilization of water as a substitute for asparagus fructans extract was the control group.

#### 2.14.2. Paralysis Assay in GMC101 Strain

Transgenic *C. elegans* strain GMC101 was synchronized by treating gravid hermaphrodites with alkaline hypochlorite solution [27]. The eggs obtained were shaken (120 rpm) at 16 °C overnight in M9 buffer (22 mM KH_2_PO_4_ 42 mM Na_2_HPO_4_ 86 mM NaCl 1 mM MgSO_4_) to allow hatching. The next day, L1 animals (200 L1/plate) were transferred to fresh NGM agar plates (60 mm diameter) seeded with OP50 and treated with 100 µL of fructans extract (168 µg/mL) or with water (control) and allowed to reach L4 stage at 16 °C. The synchronized L4 was transferred to fresh NGM plates spotted with OP50 containing either fructans extracts or water (100 µL) (*n* = 60 worms/group, 30 worms/plate). The worms were incubated at 25 °C to induce amyloid-β aggregation for 16 h. The amyloid-β peptides are aggregated in the muscle cells of GMC101 strain, which causes paralysis in the mutants. The number of paralyzed worms was scored under the microscope. To identify the paralysis, each worm was gently touched with a platinum wire. Worms were considered to be paralyzed if they moved neither spontaneously nor in response to three prods on the head, at least one time their full body length.

#### 2.14.3. Determination of Progeny Production in *daf-2(e1370)*

Synchronized L1 worms (200 L1/plate) were transferred to fresh NGM agar plates (60 mm diameter) spotted with OP50 containing either 100 µL fructans extracts (168 µg/mL) or water (control) and were allowed to develop to the late L4 stage (around 2.5 days) at 16 °C. This low temperature was chosen to avoid dauer formation in the temperature-sensitive *daf-2(e1370)* mutant. At L4 stage (around 2.5 days later), thirty worms were transferred to small fresh NGM agar plates (35 mm diameter) spotted with OP50, also with or without 50 µL of fructans extracts (168 µg/mL) (*n* = 30 worms/group, 1 worm/plate), the worms were incubated for 72 h at 25 °C. The progeny production was determined by quantifying the number of progenies hatched out of eggs. This assay was performed at least three times independently.

### 2.15. Statistical Analysis

All samples were analyzed at least in triplicate. To assess the differences among samples, a multiple-sample comparison was performed using the Statgraphics^®^ Plus program Version 2.1. The level of significance was *p* < 0.05.

GraphPad Prism 9 (Version 9.0a) was used to analyze the data from *C. elegans*. Experiments yielding quantitative data for statistical analysis were performed independently at least three times, all with similar results. Micrograph images shown in the figures are representative of three independent experiments, all with similar results for progeny production assay and the average of three independent experiments for paralysis assay.

## 3. Results and Discussion

### 3.1. Asparagus Inulin Yield and Chemical Composition

As described in the Methods section, 500 g of roots and rhizomes from asparagus plants were treated with 4 L of water and then a second extraction was carried out with 2 L of water. Both filtrates were pooled (4.8 L) and subjected to a purification step by adsorption chromatography. This step was necessary because asparagus roots are also a potential source of steroidal saponins [21,28,29,30], which must be removed from root extracts. This saponin extract also has its own interest for its technological and functional characteristics [28,30,31]. After purification, fructan-containing fractions were concentrated under a vacuum and freeze-dried. The complete process was repeated eight times at least and an average yield of 17.51 ± 1.95 g/100 g fresh roots was determined (48.57% dry weight). The obtained residue (PAF) was a dry light-brown powder that was compared with other commercial inulins in order to know its possible technological and dietetic applications.

**Figure 1:**
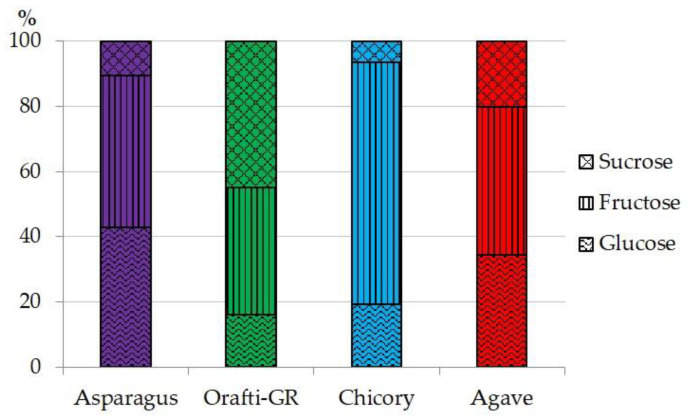
Percentual composition of simple sugars in the different fructan samples.

PAF and the commercial samples were analyzed for their content in moisture, simple sugars, ash, protein and fructan and the results are presented in Table 1. Although the last step for PAF obtaining was freeze-drying, 9% of its moisture remained. This content was the highest of the four samples analyzed, with the inulin from chicory at 6%, and that from agave and Orafti^®^GR at 4.6%. The high content of moisture could be related to the protein content in PAF, as the good water-holding capacity of proteins is widely known [8]. The amounts of simple sugars (glucose, fructose and sucrose) are very important, especially from a dietetic point of view. Orafti^®^GR had the lowest contents and the inulin from chicory showed the highest. PAF had twice the sugar content of Orafti^®^GR, around 4%, but half the amount of sugar was quantified in the other commercial samples. The composition of simple sugars is presented in Figure 1. The inulin from asparagus and agave were in similar proportions with similar amounts of glucose and fructose and only small amounts of sucrose. Sucrose was the major component in Orafti^®^GR, and fructose was the most abundant in the sample from chicory. Considering fructans as healthy food ingredients, it is important to maintain the level of simple sugar as low as possible, as fructans are often used as sugar replacements in diabetic and low-calorie foods [32]. Even more important than sugar content is the fructan content in the PAF and other commercial samples. Two groups could be clearly established from the four samples analyzed: Orafti^®^GR and inulin from agave, which have nearly 80% fructan content, and PAF and inulin from chicory, which were around 60%. From a dietetic point of view, the ratio of fructan/simple sugar should be as high as possible. This value was the highest for Orafti^®^GR (39.25), followed by PAF and agave (13.83 and 11.44, respectively), and finally chicory (6.95) inulins.

Therefore, we have a 48.57% yield on asparagus root dry weight basis and 58% fructans in PAF. With these percentages, we can assume that asparagus roots contain around 28% fructans on a dry weight basis. Chicory roots, Jerusalem artichoke tubers and agave stems, the usual industrial sources of fructans, have a much higher yield of fructans as expressed in dry weight: 63–80% for chicory [33,34]; 65–84% for Jerusalem artichoke [35,36], and 38–73% for agave [37,38]. However, other published works on agricultural by-products such as fructan sources reported lower amounts for this component, e.g., 16–28% in artichoke waste [39], around 5% in onion by-product [40] and 1.5% in cashew apple [41]. The extract obtained from artichoke waste contained 60–80% fructans, and that of onion, around 10%. If additional purification steps were applied to PAF, its fructan percentage would increase. Ion exchange chromatography can be implemented for ash and protein removal [8], and also treatment with calcium hydroxide and phosphoric acid for deproteinization [42]. Considering these facts, asparagus roots and rhizomes are similar to artichoke waste, the best agricultural by-product described as a fructan source in the bibliography, and its industrial exploitation could increase the income of asparagus spear producers.

Phenolics are also present in PAF (Table 2) but not in the other samples and, as a consequence, PAF also showed antioxidant activity. Chelidonic acid was the major component, followed by caffeic acid and caffeoyl-glycoside. Plants containing chelidonic acid are widely used in folk medicine as anti-inflammatory agents [43,44,45], with therapeutic potential for allergic disorders [43], mild analgesic, antimicrobial, oncostatic and sedative [44], and even in ethnoveterinary uses [46]. Caffeic acid is commonly known as a natural healing agent with medicinal properties because it possesses antioxidant, anti-inflammatory, anticancer, and neuroprotective properties [47]. Therefore, the phenolic compounds present in PAF cannot be considered contaminants to be removed but as bioactive constituents with valuable activities.

### 3.2. Degree of Polymerization of PAF and Commercial Inulins

**Figure 2:**
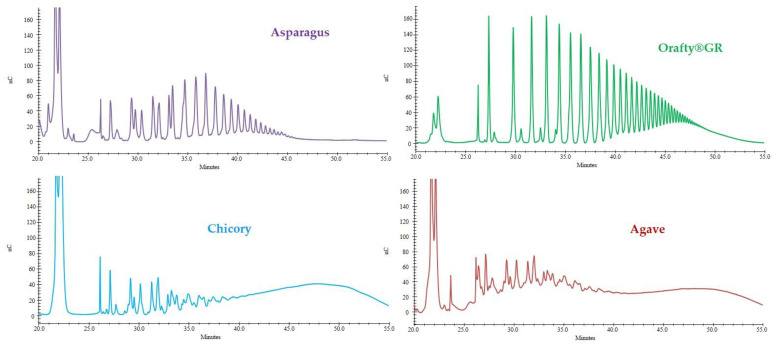
HPAEC profiles of the different fructan samples: asparagus fructan, Orafti^®^GR, chicory inulin from SaludViva, and agave inulin from Tentorium Energy S.L.

The elution profiles of the different fructans analyzed by HPAEC are presented in Figure 2. PAF showed similar characteristics to Orafti^®^GR, and inulins from chicory and agave showed almost identical profiles. Orafti^®^GR, which is obtained from chicory roots, is widely used in food technology as a fat replacer in the formulation of baked goods, breakfast cereals, candy and chocolates, soups and sauces, dairy and meat products, etc. [48] without losing their creamy mouthfeel and providing low caloric content. Chicory fructans are of the inulin-series, linear polymers of fructose linked to the fructose unit of a sucrose molecule by β(2→1) linkage [49]. In Asparagales (asparagus and agave), apart from the inulin series, there is also a variable percentage of inulin-neo series (fructose is bonded β(2→6) to glucose in sucrose and β(2→1) on the fructose residue). In these plants, a great variety of isomers were described [15,50,51,52]. The case of agave is even more complex because this crop survives in a severe drought environment, which boosts the synthesis of all kinds of fructans [49].

In the present study (Figure 2), fructans from asparagus roots contained polymers of up to 25 sugar units but also a variety of iso-isomers were identified, as previously reported [15]. In Orafti^®^GR, polymers of higher molecular weight were found (up to 40 DP). In this case, the presence of iso-isomers was reduced to a minimum. The commercial inulins from chicory and agave looked very similar and were identified as oligomers of up to 10–11 DP. In fact, they should be labeled as “oligofructose” or “fructooligosaccharides” (FOS) rather than “inulin” because the term “inulin” refers to polymers with DP > 10 [53].

DP is a very important fructan characteristic because it marks the possible applications of the product. Oligofructose with DP ≤ 10 have some degree of sweetness (30–65% compared to sucrose), they are very soluble in water, not texturizing [54], but are easily and quickly fermented by colonic microbiota, with high prebiotic activity. However, the sweetness of inulins with DP > 10 is very low (0–30%), they produce viscous solutions in water [48], and are fermented more slowly in the gut. When ingested, oligofructose is fermented mainly in the proximal zone of the colon. On the contrary, inulin arrives at the distal portion where most chronic diseases occur and its beneficial prebiotic activity can be performed [55]. It is clear that fructan DP influences not only its technological applications but also its prebiotic characteristics.

### 3.3. Physicochemical Characteristics of PAF and Other Commercial Inulins

**Figure 3:**
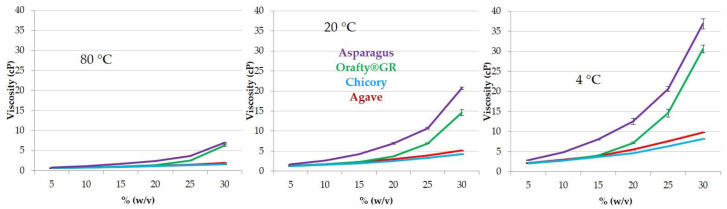
Viscosity patterns of the different fructan samples at different concentrations (5–30% *w*/*v*) and temperatures (80 °C, 20 °C, and 4 °C).

As mentioned above, viscosity is a key property for the use of inulins as fat replacers and it is directly linked to DP. In Figure 3, the viscosity of inulins from different origins at several concentrations and temperatures is presented. At the three tested temperatures, the behavior of samples was similar: in all cases, PAF and Orafti^®^GR showed close values, although PAF solutions were always the most viscous ones. Inulin from agave and chicory presented significant differences only at 4 °C. It is very clear that the correlation between DP and viscosity is that the lower the DP, the lower the viscosity.

Both factors, temperature and concentration, had a great influence on sample viscosity. For all samples, the decrease in temperature from 80 to 4 °C increased viscosity between 300 and 500 times (depending on concentration, lower concentrations corresponded to lower increases). However, the increase in concentration from 5 to 30% at the three temperatures resulted in differences among the samples. In chicory and agave, the viscosity increased 250–280 times at 80 °C, 340–380 times at 20 °C, and 380–440 times at 4 °C. When PAF and Orafti^®^GR were studied, greater increases were detected: 900–1000, 1000–1200 and around 1400 times, respectively, at 80, 20 and 4 °C. The effect of concentration was higher in samples with high DP than in those with low DP and was obviously also higher at lower temperatures.

Although the PAF extract had lower fructan content with lower DP than Orafti^®^GR, its viscosity results were always higher. This fact could be due to the presence of proteins in PAF (17.5%). Interactions such as charge–dipole, charge-induced dipole, and hydrogen bonds are mainly responsible for the viscosity of protein solutions [56]. Furthermore, it is widely known that the addition of sugars or polyols acts as a protein stabilizer by increasing the viscosity of the solution [57,58]. The presence of fructans and proteins in PAF resulted in more viscous solutions when compared to Orafti^®^GR. The creamy texture of both products at medium–high concentrations (>20%) and medium–low temperatures (<40 °C) allows for their application as fat replacers in multiple food formulations (ice creams, dressings, dairy products and juices) [59,60,61].

The results obtained for pH, OHC and color are presented in Table 3. There are significant differences in pH among the samples, between 4.6 and 6.4 units. These values are in the same range as another published previously [8,38]. OHC bundled the samples into groups: Orafti^®^GR and agave (around 79 g oil/100 g sample), and PAF and chicory with a significantly higher OCH (about 94 g/100 g). This fact could be related to the purity of the samples because those with lower purity (near 58% in fructans, Table 1) showed higher OHC results. The presence of other compounds such as proteins, simple sugars or phenols was likely to improve this property. Higher values were found for inulin isolated from Jerusalem artichoke tubers [62], up to 145 g oil/100 g product.

The color of PAF was clearly different from that of the other fructan sources (Table 3) because the asparagus extract was pale brown. The L* (lightness) value decreased and the b* value (yellowness) went up. The same happened for other unpurified fructans from natural sources such as Jerusalem artichoke tubers [62], *Asparagus falcatus* and *Taraxacum javanicum* plants [8].

### 3.4. FT-IR Spectrum for Different Inulins

**Figure 4:**
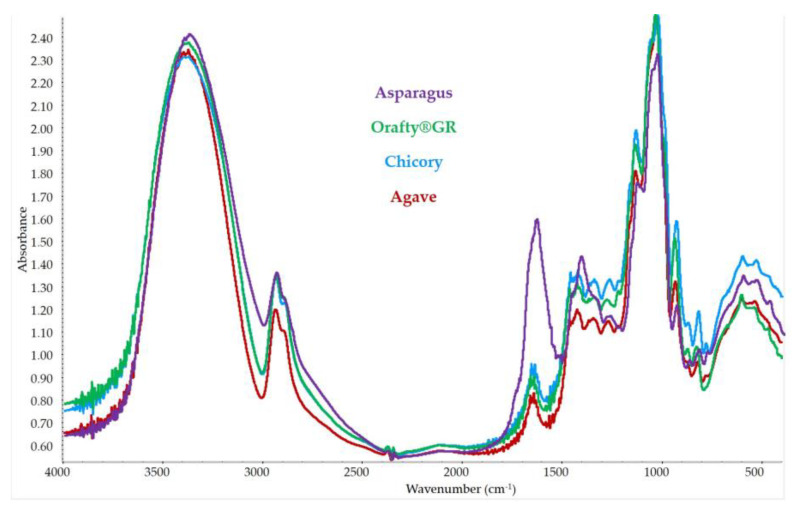
FT-IR spectrum of the different fructan samples.

In Figure 4, the spectra of the different samples are presented. They presented the typical bands described previously for inulin [63]. The broad band between 3600 and 3200 cm^−1^ was due to the stretching of OH- groups from both carbohydrate and phenolic groups. The lower band at 3000–2800 cm^−1^ is attributed to C-H vibrations. In the spectral region of 1500–800 cm^−1^, there is another group of bands related to carbohydrates: 1500–1250 cm^−1^ for C-C-C stretching, and 1250–800 cm^−1^ for C-O stretching, C-O-H and C-O-C bending [38]. PAF showed a more intense band in this zone that could be due to bending and stretching vibrations in aromatic rings [64].

There was another band in the spectral region of 1700–1500 cm^−1^, where PAF looked different. A band was present in all samples in this zone but that of PAF was much higher than that of the other ones. In this zone, there was a band of C=O stretch, which is characteristic of inulin [8]. However, in this zone, there were bands assigned to proteins [38] (amide I and amide II vibrations) and to aromatic compounds (C-H and C=C-C aromatic bond stretching) [65]. The presence of both groups of compounds in PAF could explain this high-intensity band at 1700–1500 cm^−1^. These results supported the presence of purified fructans in the root extract of asparagus plants. The comparison of the PAF spectrum with those of other commercial fructans confirmed that all the samples had very similar compositions but with higher amounts of proteins and phenolics in asparagus roots.

### 3.5. Prebiotic Activity of Inulins In Vitro

**Figure 5:**
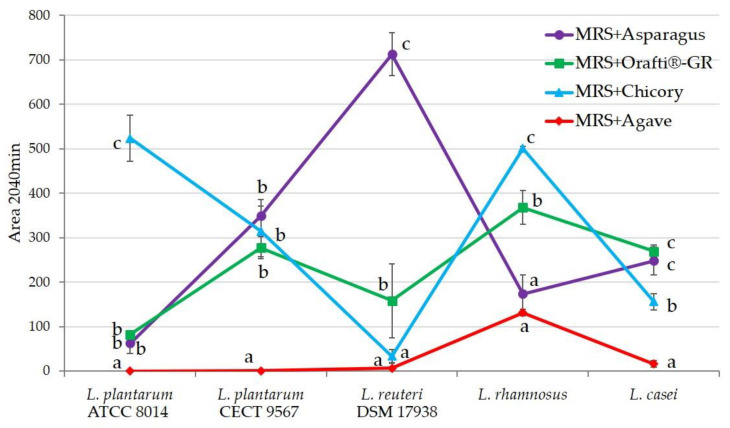
Area under the growth curve for the different inulins studied and several *Lactobacillus* strains. The bar for each value indicates the standard deviation. All analyses were performed at least in triplicate. Values bearing the same letter are not significantly different at the 5% level, as determined by the Duncan multiple range test.

The obtained results are presented in Figure 5. Each *Lactobacillus* strain showed different behavior against the different fructans assayed. In general, fructans from agave were hardly fermented, only *L. rhamnosus* showed some capacity. The other three samples demonstrated prebiotic activity depending on the strains. *L. reuteri*, which was the strain with the lowest fermentation capacity, also showed the highest one on PAF. Orafti^®^GR was mildly fermented by almost all the strains, except for *L. plantarum* ATCC 8014. Fructans from chicory presented a very good fermentability with *L. plantarum* ATCC 8014 and *L. rhamnosus*. PAF showed excellent results with *L. reuteri*, low fermentability against *L. plantarum* ATCC 8014, and moderate against the other three strains.

This variability in the activity of the different prebiotic/probiotic pairings was previously described. Working with chicory inulin and its hydrolysis products (oligofructose), Roberfroid et al. [55] discussed that *Bifidobacterium infantis* optimally fermented both substrates, *Bifidobacterium animalis* only oligofructose, and *Bifidobacterium bifidum* any of them. Similar results were found when fructans from *Agave angustifolia* [38] and artichoke waste [39] were assayed against several *Lactobacillus* and *Bifidobacterium* species.

### 3.6. Effect of Fructans Extracts against Aβ-Induced Paralysis

**Figure 6:**
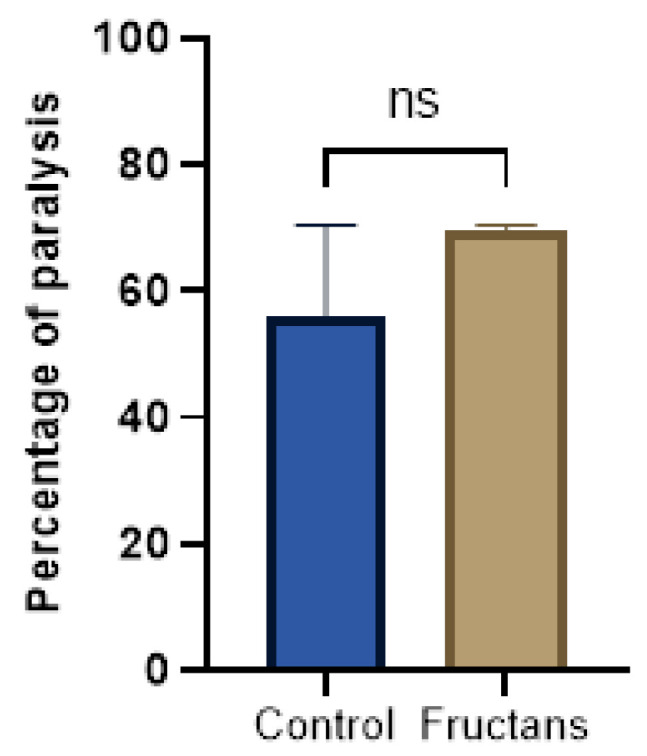
Percentage of paralyzed worms at a non-permissive temperature (25 °C). Fructans extracts (168 µg/mL) do not ameliorate the paralysis phenotype of GMC101 nematode model of Alzheimer’s disease. The percentage of paralyzed worms at a non-permissive temperature (25 °C) in non-treated (blue bar) and treated (brown bar) GMC101 strain was presented. Results are expressed as mean ± SD. ns represents no significant effect of fructans compared to the control using two-tailed *t*-test. Final data represented the average of three independent assays.

A paralysis test was performed to analyze any potential effect of the fructan extracts on the transgenic *C. elegans* model of Alzheimer’s disease. For this purpose, the GMC101 strain was used. This strain expresses the human amyloid-β peptide in muscle cells in a temperature-inducible way, which leads to a paralyzed phenotype. L1 GMC101 was grown on nematode growth medium with or without fructan extracts at 16 °C until they reach the L4 stage and then shifted to 25 °C to induce Aβ aggregation. Figure 6 illustrates the percentage of paralyzed worms at a non-permissive temperature (25 °C). The result showed that asparagus fructans at a concentration of 168 µg/mL do not ameliorate the paralysis phenotype.

The broad variety of chemical compounds found in plant natural products, which can be employed as sources for new Alzheimer’s disease therapies, may perhaps play a crucial role in drug discovery. Numerous natural compounds’ abilities to reduce amyloid-β toxicity were investigated in several *C. elegans* strains. Using the same strain of our study, a large variety of plant products was evaluated in these models which showed potential in reducing Aβ-induced paralysis such as triterpenoids and flavonoid glycosides from *Dillenia suffruticosa* leaves [66], flavonoids from sour Jujube seed [67], Danshen (*Salvia miltiorrhiza*) water extract [68], flavonoid glucosides from *Ziziphus jujuba* seeds [69] and so on. Nevertheless, few studies have explored the effect of soluble polysaccharides on toxicity and aggregation of amyloid-*β* in the transgenic *C. elegans* model of Alzheimer’s disease [70,71].

### 3.7. Effect of Fructan Extracts on the Fertility of the Mutant daf-2(e-1370)

**Figure 7:**
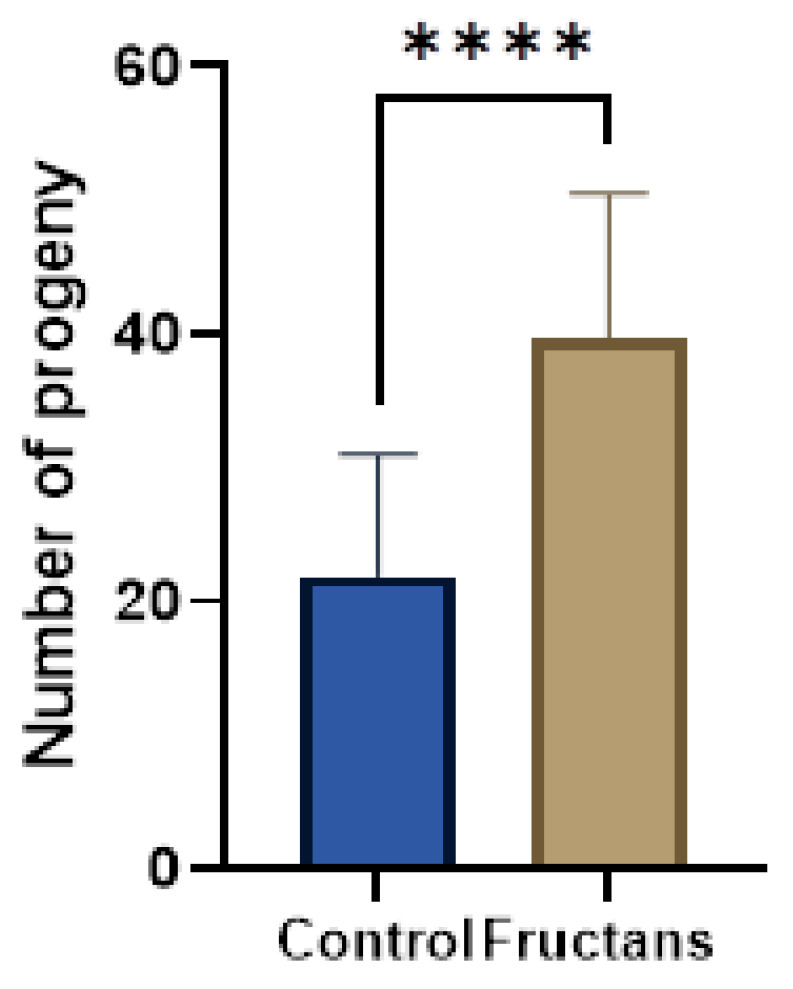
Progeny of *daf-2(e1370)* strain in presence or absence (control) of asparagus fructans. Asparagus fructan extracts (168µg/mL) enhance fertility of the mutant *daf-2*(*e-1370*). In the X-axis, the blue bar corresponding to *daf-2*(*e-1370*) without fructan extract treatment and the brown bar corresponding to *daf-2*(*e-1370*) treated with 168 µg/mL of fructan extract. The Y-axis indicates the average total number of progenies per worm produced during 72 h. The total number of progeny produced increased significantly following asparagus fructans extracts treatment. Graphs represent mean ± SD *n* = 30. **** represents *p* < 0.001 using two-tailed *t*-test. Two more replicates with similar results were carried out to confirm these effects (Supplementary Material).

A crucial regulator of growth, development, and longevity is the insulin/IGF-1 signaling (IIS) pathway, which has been conserved throughout evolution [72]. In humans, high levels of insulin signaling are linked to tumor growth and cancer, whereas low levels of insulin signaling cause insulin resistance and diabetes [73]. Given its consequences, diabetes mellitus poses a serious risk to human health on a global scale [74]. All over the world, it is one of the top five most common causes of death [75]. Hyperglycemia and glucose intolerance lead to the development of a group of metabolic diseases known as diabetes mellitus. There are two types of diabetes mellitus that are frequently recognized. Insufficient insulin release from the pancreatic cells characterizes type-1 diabetes, whereas the emergence of insulin resistance in the body characterizes type-2 diabetes [76]. An excellent animal model for studying insulin-like signaling is the nematode *C. elegans*. Reduced or partial loss of function on the insulin pathway in *C. elegans* can alter stress responses or affect life span, among other pleiotropic effects [77]. Low or non-existent fertility in *C. elegans* were linked to mutations in the insulin signaling pathway [78]. This pathway is evolutionarily conserved in all metazoans and similarly to *C. elegans*, other organisms, including humans, also showed a correlation between reduced fertility and deficiencies in the activity of the insulin signaling pathway [79]. In the present work, we selected a *C. elegans* mutant strain named *daf-2*(*e1370*) affected in the human homolog of the insulin and insulin-like growth factor-I (IGF-I) receptors, which exhibits a severe reduction in fertility and altered embryonic development [80]. To prove the effects of asparagus fructans extracts on the fertility of *daf-2*(*e1370*), progeny numbers were quantified compared to control and represented in Figure 7 and Figure S1.

The results showed that asparagus fructan extracts double the progeny in this mutant, presenting a significant increase in the average descendent per worm (40 vs. 20). To the author’s knowledge, no research has been carried out on the improvement of *daf-2*(*e1370*) fertility by plants source fructans. In a previous work [81], the wild-type *C. elegans* treated with 100 µg/mL fructans from an exopolysaccharide secreted by *Weissella cibaria* lived 64% longer than the control under juglone oxidative stress. The forkhead transcription factor DAF-16, which is crucial for the insulin pathway, mediates this rise in oxidative stress resistance. Under normal conditions, the insulin pathway keeps DAF-16 inactive and outside of the nucleus. When there is an impairment of the insulin pathway, DAF-16 goes to the nucleus to control most of the insulin pathway-related phenotypes. Consequently, a mutation in *daf-16* can suppress most of the known insulin pathway mutants’ phenotypes, including *daf-2(e1370)* infertility [82]. Paradoxically, the effect of fructans from *Weissella cibaria* and those from asparagus seems to be opposite in their relation with *daf-16*, but as far as we are looking at different phenotypes and different types of fructans, this point needs further investigation.

Insulin affects blood glucose levels in humans but it also plays a significant role in the reproductive system by interacting with insulin and insulin-like growth factor receptors in the brain and testis. Studies have shown that insulin plays crucial roles in spermatogenesis, prostatic growth, Sertoli cell proliferation and differentiation, testicular descent, and sexual behavior [83,84,85,86,87]. Infertility and impaired testis growth and maturation may also result from the absence of insulin receptors in GnRH neurons or Sertoli cells [88,89]. In addition, decreased levels of gonadotropins and testosterone, altered androgen-to-estrogen ratios, impaired semen parameters, and erectile dysfunction occurs in obese men with insulin resistance [90,91]. Interestingly, recent studies showed that inulin-type fructans improve glycemic control and moderate insulin resistance and lipid metabolism in prediabetic and type 2 diabetic patients [92,93].

## 4. Conclusions

Fructans are commercially obtained from chicory, agave and Jerusalem artichoke, depending on the production zone (Europe, South America and North America, respectively). However, asparagus underground organs are agricultural by-products that could be considered sources of fructans. As discussed in this work, a purified extract of asparagus roots could be regarded as a fructan extract with technological potential. Its composition and physicochemical characteristics are similar to other commercial fructans and show proven prebiotic activity. Therefore, asparagus fructans could be used as a fat replacement for medium–low-temperature foods (salad dressings, ice cream, pastry fillers, etc.) with its low sugar and calorie content. Due to its molecular weight and prebiotic activity, its use could provide in addition beneficial effects on colon proximal and distal portions. The preliminary results on its therapeutic possibilities opened a broad field for possible health applications. Changing the idea of asparagus roots as a troublesome agricultural by-product for that of a valuable feedstock could be a great goal for asparagus cultivation sustainability and a successful endeavor from the point of view of the circular economy.

## Figures and Tables

**Table 1:** Chemical composition of inulin from different sources.

	Moisture	Simple Sugars	Ash	Protein	Fructans
Asparagus	9.00 ± 0.13 ^c^	4.19 ± 0.43 ^b^	6.69 ± 0.06	17.50 ± 0.83	57.94 ± 0.41 ^a^
Orafti-GR	4.61 ± 0.37 ^a^	1.99 ± 0.01 ^a^	t	n.d.	78.11 ± 0.51 ^b^
Chicory	6.00 ± 0.57 ^b^	8.48 ± 0.59 ^d^	t	n.d.	58.96 ± 1.44 ^a^
Agave	4.60 ± 0.11 ^a^	7.05 ± 0.39 ^c^	t	n.d.	80.65 ± 2.47 ^b^

All analyses were performed at least in triplicate. The results are presented as mean ± standard deviation. Means bearing the same letter are not significantly different at the 5% level, as determined by the Duncan multiple range test. n.d.: not detected. t: traces.

**Table 2:** Phenolic composition and antioxidant activity of asparagus fructan extract. TE: Trolox equivalents.

**mg/g Asparagus Fructan**	
Chelidonic acid	1.13 ± 0.07
Caffeic acid glycoside	0.30 ± 0.00
Caffeic acid	0.64 ± 0.03
p-Coumaric acid	0.07 ± 0.00
t-Ferulic acid	0.11 ± 0.01
Total	2.25 ± 0.03
**μmols TE/g asparagus fructan**	
Antioxidant activity	43.62 ± 3.16

All analyses were performed at least in triplicate. The results are presented as mean ± standard deviation.

**Table 3:** pH, oil holding capacity (OHC) and color of the different fructan samples.

			Color		
	pH	OHC	L*	a*	b*
Asparagus	6.45 ± 0.01 ^d^	93.63 ± 1.41 ^b^	50.5203	3.9032	17.3581
Orafti^®^GR	6.04 ± 0.02 ^c^	78.46 ± 3.01 ^a^	88.8906	−0.3873	2.6834
Chicory	5.12 ± 0.03 ^b^	95.15 ± 4.76 ^b^	86.9714	−0.1935	3.039
Agave	4.59 ± 0.23 ^a^	79.93 ± 2.78 ^a^	88.1635	−0.6182	4.652

All analyses were performed at least in triplicate. The results are presented as mean ± standard deviation. Means bearing the same letter are not significantly different at the 5% level, as determined by the Duncan multiple range test.

## Data Availability

Data are contained within the article or the Supplementary Material.

## Supplementary Materials

The following supporting information can be downloaded at: https://www.mdpi.com/article/10.3390/foods12010081/s1, Figure S1: Asparagus fructans extracts enhances fertility of the mutant daf-2(e-1370) (168µg/mL). The total number of progeny produced increased significantly following asparagus fructans extracts treatment. A: the second independent experiment and B: the third independent experiment. Graphs represent mean ± SD *n* = 30. ** *p* ≤ 0.01, *** *p* ≤ 0.001 using Two-tailed *t*-test.
