# Supplementary material for: Asparagus Fructans as Emerging Prebiotics
Source: Foods. 2022 Dec 23;12(1):81. doi: 10.3390/foods12010081 (PMC9818401; doi:10.3390/foods12010081)
Supplement: Supplementary file 1 [file foods-12-00081-s001.zip › foods-2074523-supplementary.pdf]

Supplementary Material

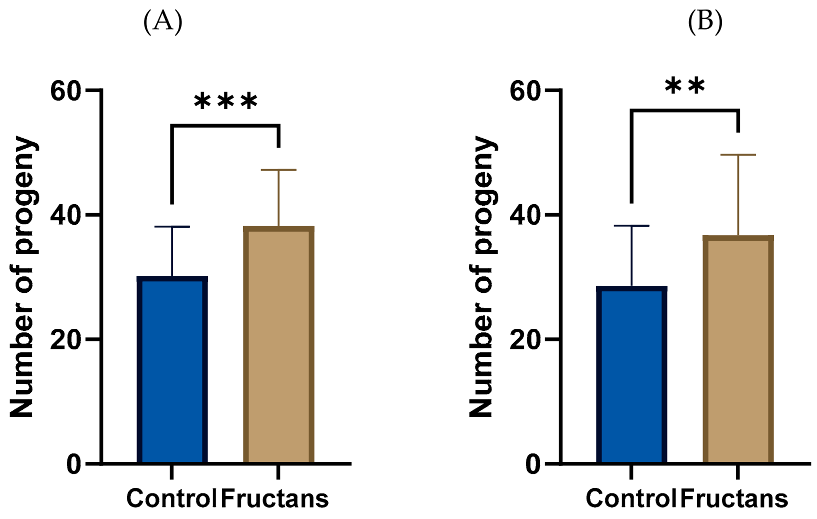

Figure S1. Asparagus fructans extracts enhances fertility of the mutant *daf-2(e-1370) (168µg/mL)*. The total number of progeny produced increased significantly following asparagus fructans extracts treatment. **(A):** the second independent experiment and **(B):** the third independent experiment. Graphs represent mean ± SD n = 30. ** $p \leq 0.01$, *** $p \leq 0.001$ using Two-tailed *t*-test.
